# Prothrombotic Rebound After Discontinuation of Direct Oral Anticoagulants Therapy: A Systematic Review

**DOI:** 10.3390/jcm13216606

**Published:** 2024-11-03

**Authors:** Marta Frydrych, Maciej Janeczek, Agata Małyszek, Kamil Nelke, Maciej Dobrzyński, Marceli Lukaszewski

**Affiliations:** 1 Anaesthesiology and Intensive Care Unit, Sokolowski Specialist Hospital in Walbrzych, Sokolowski 4, 58-309 Walbrzych, Poland; marceliluk@gmail.com; 2 Department of Biostructure and Animal Physiology, Wroclaw University of Environmental and Life Sciences, Kożuchowska 1, 51-631 Wroclaw, Poland; agata.malyszek@upwr.edu.pl; 3 Maxillo-Facial Surgery Ward, EMC Hospital, Pilczycka 144, 54-144 Wroclaw, Poland; kamil.nelke@gmail.com; 4 Academy of Applied Sciences Angelus Silesius in Walbrzych, Health Department, Zamkowa 4, 58-300 Walbrzych, Poland; 5 Department of Pediatric Dentistry and Preclinical Dentistry, Wroclaw Medical University, Krakowska 26, 50-425 Wroclaw, Poland; maciej.dobrzynski@umw.edu.pl

**Keywords:** DOACs, dabigatran, rivaroxaban, apixaban, edoxaban, prothrombotic rebound, hypercoagulability, antithrombotic therapy

## Abstract

**Background/Objectives**: The practice of holding anticoagulation is a relatively common approach, with the aim of either preventing excessive bleeding in preparation for surgical procedures or managing acute bleeding episodes. **Methods**: To assess the relationship between the discontinuation of direct oral anticoagulants (DOACs) therapy and the condition of hypercoagulability, a systematic review of the literature was conducted, following PRISMA guidelines, in PubMed/MEDLINE, Cochrane, and Google Scholar. These databases were searched for all publications that described a rebound phenomenon or hypercoagulability state after DOACs discontinuation. **Results**: A total of 1494 articles were selected from searched databases, and 29 were eligible. A final total of 16 case reports and 14 original research articles were subjected to analysis. **Conclusions**: The results of this study indicate that the cessation of DOAC therapy may be associated with an increased risk of thrombotic events. More studies are required to ascertain whether DOACs treatment cessation can be linked to rebound phenomena associated with thromboembolic events. This will provide the data needed to determine the incidence and risk of this phenomenon.

## 1. Introduction

The perspective on anticoagulation has changed significantly with the availability of direct oral anticoagulants (DOACs), previously known as new oral anticoagulants (NOACs) or target-specific oral anticoagulants (TSOACs) [1]. DOACs include direct thrombin inhibitors (dabigatran) and direct factor Xa inhibitors (rivaroxaban, apixaban, and edoxaban). Patients undergoing treatment with DOACs do not require coagulation monitoring, except for patients with renal or hepatic impairment, suspected overdose, serious bleeding or thrombotic events, and prior to emergency surgery [1].

To minimize the risk of bleeding and thrombotic events, anticoagulants should be carefully managed before invasive procedures [1]. The withdrawal of DOACs therapy is a relatively common clinical practice, typically undertaken with the intention of either preventing excessive bleeding in preparation for surgical procedures or managing acute bleeding episodes. Because of the rapid onset of action and short half-life of DOACs, there is less need to switch to parenteral anticoagulant therapy in the perioperative period, although bridging with heparin should be considered in patients at high risk of thrombosis [1].

The sudden cessation of DOAC therapy is hypothesized to potentially result in a shift in the equilibrium towards a prothrombotic state. The precise mechanism of rebound phenomena remains unclear. One potential explanation is that a reduction in plasma concentrations of rivaroxaban following the cessation of therapy may result in the “unmasking” of thrombus-associated prothrombinase [2].

It remains uncertain as to whether the cessation of treatment with DOAC precipitates a paradoxical prothrombotic state due to rebound. There is preclinical evidence that a rebound prothrombotic state may occur following the short-term cessation of dabigatran treatment [3]. There was no evidence of recurrent ischemic incidents following a change from dual therapy to DOAC monotherapy [4]. 

DOACs are widely prescribed and used in clinical practice, primarily in patients with atrial fibrillation or venous thromboembolism. It is crucial to address existing research gaps regarding the safety of DOAC discontinuation.

The research project was designed to achieve a comprehensive review of all documented cases of rebound phenomena following the cessation of DOAC therapy. This was complemented by an extensive and systematic review of the available literature on the subject.

## 2. Methods

This study was conducted according to the PRISMA statement [5].

### 2.1. Search and Study Identification

A search of the literature was conducted in PubMed/MEDLINE, Cochrane, and Google Scholar. All searches were performed on 17 August 2024. The search strategy was based on the Boolean method, allowing the researchers to limit the results to studies that met the pre-defined criteria for relevance. The following query was used for the research: ((discontinuation) OR (cessation) OR (stopping taking) OR (stop taking) OR (discontinuing) OR (withdrawal)) AND ((DOAC) OR (direct oral anticoagulants) OR (NOAC) OR (new oral anticoagulants) OR (target-specific oral coagulants) OR (TSOAC) OR (antithrombotic therapy) OR (dabigatran) OR (rivaroxaban) OR (apixaban) OR (edoxaban)) AND ((rebound) OR (rebound phenomena) OR (prothrombic rebound) OR (prothrombic state) OR (hypercoagulability)). All duplicates were excluded from the review process.

Two authors (MF and ML) conducted an independent screening of titles and abstracts to ascertain their relevance. The full texts were examined in accordance with the established inclusions and exclusions criteria. The resolution of the disputed articles was reached through discussion among all authors.

### 2.2. Selection Criteria

In order to be included in the present review, an article must meet the following criteria: (1) be published in the English language; (2) have an available abstract; (3) include human subjects with no limitation to age; (4) include relevant data on the relationship between DOACs therapy cessation and rebound phenomena. No restrictions were placed on the publication date, as we assumed that the results would be limited in number. The reference lists of the selected studies were examined to identify any additional relevant studies.

The following criteria were used to exclude studies from the review: (1) no abstract; (2) no access to full text; (3) including laboratory animals; (4) laboratory study; (5) uninterrupted DOACs therapy and thrombotic event; (6) discontinuation of non-DOACs; (7) reversal agents; (8) hemorrhagic event during taking DOACs; and (9) reviews, letters, book chapters, reports, conference materials, commentaries, expert opinions.

### 2.3. Data Extraction

The author responsible for initial extraction of data from each included study used predesigned table forms on Microsoft Word. The second author then verified the results. The remaining authors resolved any discrepancies between the authors.

## 3. Results

**Figure 1:**
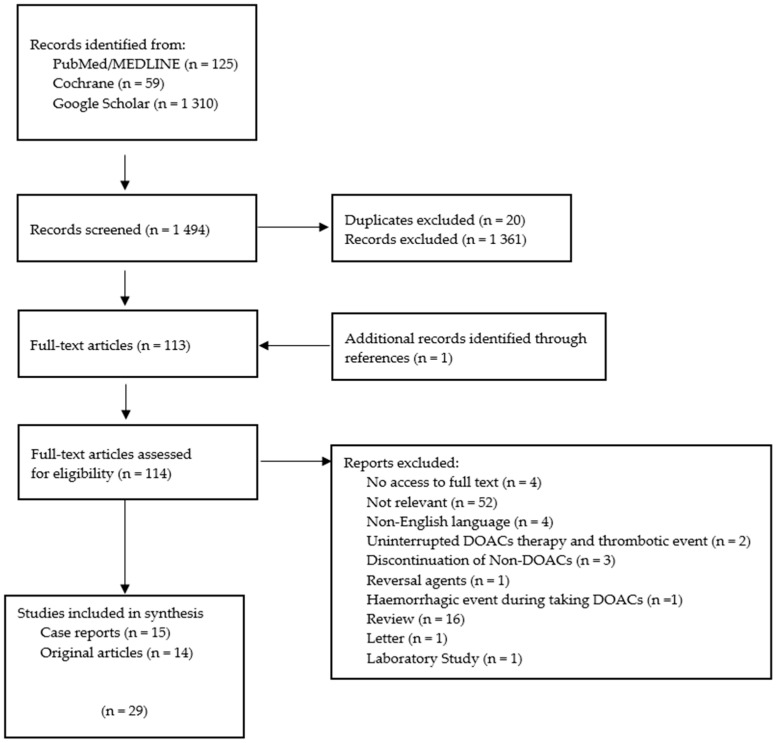
The PRISMA flow diagram illustrates the process of selecting the journal articles included in the systematic review.

As illustrated in Figure 1, the electronic databases resulted in the identification of 1494 studies, comprising 125 from PubMed/MEDLINE, 59 from Cochrane, and 1310 from Google Scholar. Following the removal of duplicates, 1474 studies were selected for further consideration, and their titles and abstracts were subjected to analysis. Following the examination of the full texts, 29 studies were identified as meeting the inclusion criteria and included in the systematic review (Figure 1).

A systematic review of 16 case reports on thrombotic events following the cessation of DOACs therapy (Table 1) identified patients aged 31–89 years, with half of them being female. The majority of patients received rivaroxaban (10 out of 16), with the remainder receiving dabigatran (4 out of 16) and apixaban (2 out of 16). The most common indication for DOAC therapy was AF, which was the primary indication in 10 out of the 16 cases. The second most common indication was VTE, which was the indication in 4 out of 16 cases. Only three patients received bridging therapy, all of which involved the use of heparin. The patients had various reasons for the cessation of their therapy, with the length of cessation ranging from 1 to approximately 122 days.

A systematic review of 14 original studies investigating the cessation of DOACs therapy and the subsequent occurrence of thrombotic events revealed a potential correlation between DOACs therapy discontinuation and an increased risk of thromboembolic events. The majority of studies included randomized controlled trials (5 of 14) and retrospective cohort studies (4 of 14). Across these reviews, the cessation was found to be associated with SSE in 9 out of 14 studies.

## 4. Discussion

A systematic review of 15 studies with 16 case reports has identified a potential association between the withdrawal of DOACs and the development of hypercoagulable state (Table 1). The compilation of 14 original studies presented here demonstrates a research gap and the absence of a definitive answer to the issues associated with the withdrawal of DOACs (Table 2).

A significant benefit of administering DOACs is the simplicity of their dosage and the fact that there is no requirement for routine monitoring of the anticoagulant effect [34]. DOACs are also considered to have stable and predictable pharmacokinetics [34]. Nevertheless, the efficacy and safety of DOACs therapy are influenced by a number of factors, including renal insufficiency, hepatic impairment and extreme body weights, which all affect DOACs pharmacokinetics [35].

Moreover, DOACs are frequently co-prescribed with other drugs due to the multi-disease nature of many patients. This co-prescription may result in interaction that alter pharmacodynamics and pharmacokinetics further affecting the therapeutic effect of the DOACs. It would appear that the principal pathways where the drug–drug interactions with DOACs occur are those involving the P-glycoprotein transporter and the cytochrome P450 enzyme complexes [36].

The primary issue with the use of DOACs is the lack of dose adjustment based on the individual characteristics of the patient, which can result under favorable conditions in the occurrence of thrombotic complications or an increase in the risk of bleeding. It is estimated that approximately 20% of patients in routine clinical practice receive an incorrect dosage, either an underdose or an overdose [37].

A further issue is the lack of comprehensive laboratory monitoring of DOACs levels, as well as the unreliability of conventional parameters for monitoring anticoagulation effect during DOAC treatment. The degree of DOAC interference with PT, INR, and aPTT is markedly contingent on the employed test assay [38]. The standard laboratory monitoring of coagulation parameters, such as TT for dabigatran, is not adequately sensitive and excludes clinically significant levels and for Xa inhibitors is not useful [39]. Calibrated ecarin-based assay and dTT may be used for measurement of dabigatran level [39]. The inability of the ECT and the ECA to meet expectations can be attributed to a failure to achieve sufficient standardization and a variability in sensitivity to dabigatran among different lots of ecarin [40]. The activity of apixaban, edoxaban, and rivaroxaban can be correctly estimated on the basis of anti-factor Xa levels [34,39]. However, the restricted capacity to undertake anti-factor Xa levels assessments represents a considerable challenge in the context of monitoring. The reliability of thromboelastography in assessing DOACs-related coagulopathy has been questioned in patients with traumatic brain injury [41].

The degree of anticoagulation achieved by DOACs is not consistent throughout the day, but rather displays varying levels of activity [38]. A patient who is taking DOAC on regular basis without any interference is within the therapeutic range between two administrations, but for example chronic DOAC overdose, drug accumulation due to acute kidney failure and suicidal overdosing may lead to much higher plasma levels [38]. Therefore, there is a possibility of an underestimation of the anticoagulation intensity if the estimation of plasma levels is based on the knowledge of the dosage and the most recent intake [38].

Despite the fact that DOACs have been designed to be prescribed without a plasma level assessment, monitoring can provide additional information that may be beneficial in certain cases [42]. This is particularly relevant in the light of the potential for prothrombotic rebound.

The decision to prescribe a particular DOACs should be guided by a comprehensive understanding of the differences in renal clearance, distribution into body tissue, and interaction potential [34]. The standard of care for patients receiving DOACs therapy includes regular checking of renal and hepatic function, along with the observation of any signs and symptoms of bleeding [35]. 

The maintenance of appropriate hemostasis is dependent upon a balanced relationship between coagulation and fibrinolysis [43]. The origin of the rebound phenomenon may be found in a disturbance of that balance. It is possible that interference with the prothrombotic system may involve the entire pro- and antithrombotic homeostasis, as observed in the case of glucocorticoids [44]. The hypercoagulable state observed in patients receiving cortisone or those with Cushing syndrome is characterized by elevated factor VIII levels, reduced fibrinolysis, and abnormal von Willebrand factor multimers composition [45].

The observed rebound in DOACs therapy is not an isolated case of the challenges associated with the discontinuation of therapies that disrupt the coagulation balance. The hypercoagulability rebound has already been described in cases of abrupt withdrawal from long-term nicoumalone therapy [46] and the cessation and subsequent resumption of warfarin therapy [47]. The temporary prothrombotic state, which is linked to an elevated risk of thrombotic incidents, has been observed in individuals who have discontinued chronic clopidogrel treatment [48]. This phenomenon is considered to be primarily caused by platelet hyperreactivity [48]. It is observed following the cessation of clopidogrel therapy, even when aspirin is continued [49]. There is a temporal relationship between the cessation of antiplatelet or vitamin K antagonist therapy and the occurrence of stroke and TIA [50].

Furthermore, the reappearance of elevated thrombotic marker levels and the rise in the number of thrombotic events have been identified in situations after stopping intravenous heparin [51]. The inability of heparin to inhibit clot-bound thrombin is a probable reason for this effect [52]. Thrombin that is bound to fibrin remains enzymatically active and forms a source of active thrombin, which can initiate the clotting process when heparin is discontinued [52]. The coagulation system undergoes rebound activation as early as a few hours after cessation of treatment, accompanied by a significant increase in thrombin production compared to the levels observed prior to or during the course of treatment [53].

The presence of DOACs has the potential to significantly interfere with the measurements of the most commonly used hemostatic parameters, including antithrombin, proteins C and S, activated protein C resistance, lupus anticoagulant, factor VIII, factor XIII, and fibrinogen [54]. Such interference may lead to misinterpretation of the results. This is a particularly noteworthy concern, as the normalization of parameters occurs four to five days following the cessation of DOACs [54]. In trying to provide an explanation of the mechanism underlying rebound phenomena, the model system was designed to demonstrate that when rivaroxaban plasma concentrations decline following the cessation of therapy, there will be an unmasking of thrombus-associated prothrombinase [2].

Thrombin is a key enzyme in the process of clotting. Dabigatran acts as a direct thrombin inhibitor, whereas apixaban, edoxaban, and rivaroxaban are direct factor Xa inhibitors, which prevent the conversion of prothrombin to thrombin. Both groups of DOACs suppress thrombin formation. A reduction in the prothrombotic capacity of the hemostatic system may result in a decline in the levels of natural anticoagulants present within the body, such as protein C and S. In this hypothetical mechanism, the rebound effect would be attributed not only to an increase in thrombin generation after DOAC withdrawal but also to a reduction in the effectiveness of natural anticoagulants.

The degree of dabigatran effect on thrombin suppression is less pronounced than that observed in warfarin; dabigatran has a reduced ability to counter the high concentrations of thrombin [55]. A reduction in the concentration of the dabigatran may result in the paradoxical thrombin generation [55]. The use of antithrombin-dependent thrombin inhibitors has been linked to the potential activation of thrombogenesis, which may occur as a result of the suppression of the thrombin-induced negative feedback system through the inhibition of protein C activation [56]. In contrast, direct factor Xa inhibitors have been demonstrated to offer a more favorable profile in terms of the reduced possibility of activation of the coagulation pathway [56].

The thrombin–antithrombin complex is regarded as a biomarker for the initial phases of coagulation activation [57]. The prothrombin fragment 1 + 2 is a marker of thrombin generation [58]. It is possible that plasma coagulation markers such as TAT and F1 + 2 may be involved in the detection or prognosis of a prothrombotic state following the discontinuation of DOACs. Nevertheless, there is a paucity of knowledge regarding the changes in these parameters after the cessation of anticoagulant therapy [59]. The identification of rebound markers would provide a foundation for the early detection of high-risk patients, thus allowing for the implementation of appropriate intervention strategies. This could include the investigation of potential bridging strategies for these patients and the determination of the optimal time to resume DOAC therapy following the cessation of treatment.

A synthesis of the case reports of prothrombotic rebound indicates that AF represents the most dominant indication for DOACs therapy in this group. Patients with chronic AF have been observed to exhibit elevated levels of von Willebrand factor and fibrinogen in comparison to patients in sinus rhythm [60]. The persistent impairment of the coagulation balance at the outset may imply that the withdrawal of DOACs in this patient cohort is more prone to result in thrombotic events.

The withdrawal of DOACs in the preoperative period necessitates consideration of the potential for the surgical procedure and the anesthesia administered to induce an imbalance in the coagulation system. Prolonged periods of immobilization, reduced fluid intake, and hemodynamic disturbances may contribute to the development of systemic hypercoagulability during surgical procedures. In line with a pharmacodynamic approach to DOACs discontinuation, rivaroxaban, apixaban, and edoxaban should be withdrawn one day before a low bleeding risk procedure and two days before a high bleeding risk procedure [61]. In patients with an estimated glomerular filtration rate (eGFR) lowered, these DOACs should be withdrawn for a period of more than 36 h prior to a procedure with a low risk of bleeding [62]. When discontinuing dabigatran before surgery, it is important to consider creatinine clearance and, depending on this and the risk of bleeding, therapy should be stopped one to four days prior to the procedure [61].

The bridging of DOACs therapy with low-molecular-weight heparin or unfractionated heparin is typically not advised during the periprocedural period [63]. In cases where the risk of thromboembolism outweighs the risk of bleeding, the choice of whether heparin bridging is an appropriate option for reducing the perioperative gap without anticoagulant therapy should be taken on a multidisciplinary basis [64].

In order to provide optimal patient safety, a comprehensive checklist covering all aspects of the specific procedure and patient characteristics that could elevate the risk of bleeding or thrombosis should be available to guide the perioperative use of DOACs [64].

### Limitations of This Study

While the number of databases searched was generally adequate, more databases could have been searched to include more articles. 

The analysis of the case reports revealed no information regarding the incidence and the prevalence of prothrombotic rebound after cessation of DOACs therapy. In cases where patients do not experience a thrombotic episode after discontinuation of therapy, neither hospital presentation nor any form of verification occurs. It would seem that not all conditions characterized by hypercoagulability carry the same level of thromboembolic risk and the notification of incidents perceived to be related to rebound leads to selective reporting. The need is for large-scale, prospective studies that evaluate the incidence of thrombotic events following various DOAC cessation protocols.

A possible limitation of this study is that not all patients from the collected cases underwent screening for congenital or acquired thrombophilia. 

In the analysis of the presented cases, patients with congenital thrombophilia were not excluded. Patients with congenital thrombophilia require indefinite anticoagulant treatment, and the discontinuation of this treatment is likely to be associated with an increased risk of recurrent thrombotic incidents in comparison to those without thrombophilia. Consequently, further investigation and additional studies are necessary to elucidate the presence of thrombotic rebound in this patient group.

Rebound phenomena have also been documented in the context of rebound of dabigatran levels in patients treated with idarucizumab, occurring a few hours after successful reversal or intermittent emergency hemodialysis in cases of massive dabigatran accumulation [65]. The incorporation of DOACs reversal agents into the exclusion criteria resulted in a narrowing of the scope of discourse surrounding this pharmacokinetic rebound phenomenon. 

## 5. Conclusions

The results of 15 studies describing 16 case reports indicate a correlation between the cessation of direct oral anticoagulant therapy and the development of hypercoagulability state. The findings of this study suggest that the discontinuation of DOACs therapy may be associated with an increased risk of thrombotic incidents.

Further clinical trials or meta-analyses are required to ascertain whether DOACs treatment cessation can be linked to rebound phenomena associated with thromboembolic events. This will provide the data needed to determine the incidence and risk of this phenomenon.

## Figures and Tables

**Table 1:** A summary of case reports in which the cessation of DOACs therapy was followed by the subsequent occurrence of a thrombotic event.

Authors & Year	Age & Sex	Comorbidities	Medications	Indication for DOAC Therapy	CHA_2_DS_2_ VASC Score	HAS-BLED Score	DOAC	Cessation Reasons	Cessation Duration (Days)	Bridging Therapy	Complications	Treatment	Results/Conclusions
Khan et al., 2024 [6]	79, F	Hypertension, hypothyroidism, state post sigmoidectomy	N/A	Chronic AF	N/A	N/A	Dabigatran	LGIB, LAAOD insertion	N/A	-	49 days after reintroduction of dabigatran LAAOD thrombus	Warfarin bridged with UFH, later apixaban for recurrent DAT	DAT despite dabigatran reintroduction
Huang et al., 2022 [7]	61, M	Over past 7 years: mesenteric vascular obstruction, cerebral venous sinus thrombosis, DVT and PE	N/A	Recurrent VTE	N/A	N/A	Dabigatran	Voluntary	N/A	-	Bilateral frontal hemorrhagic infarction with multiple cerebral thrombosis	LMWH for 14 days, then long-term dabigatran, no recurrence over 5 years	Novel *SERPINC1* mutation
Płatkowska-Adamska et al., 2022 [8]	38, M	None	None	Continuation of COVID-19 PE treatment	N/A	N/A	Rivaroxaban	End of treatment	3	-	Nonischemic CRVO with CMO	Intravitreal ranibizumab injection, enoxaparin	Hypercoagulable state
Niazi et al., 2021 [9]	60, M	Hypertension, history of PE	N/A	AF	N/A	N/A	Apixaban	Error	N/A	-	Ischemic stroke	t-PA	Hypercoagulable state
											Later that day brachial artery thrombosis	Embolectomy, heparin infusion	
											TEE: Left atrial thrombus	Bridged to warfarin with heparin infusion	
Shang et al., 2020 [10]	31, F	History of PE, congenital AT III deficiency	N/A	VTE	N/A	N/A	Rivaroxaban	Giving birth	Ca. 122	-	Intracranial venous sinus thrombosis	Urokinase thrombolysis, mechanical thrombectomy, LMWH	AT III deficiency requiring long-term OAC
Storey et al., 2019 [11]	64, F	N/A	N/A	AF	N/A	N/A	Apixaban	Coronary angiogram	5	-	Bilateral MCA occlusions	Mechanical thrombectomy	Hypercoagulable state
Göndör et al., 2017 [12]	58, M	PAD, FLD, prostatic hyperplasia, status post TIA, cholecystectomy, parotidectomy, thyroidectomy, appendectomy	Amlodipine, Valsartan, Hydrochlorothiazide, Bisoprolol, Levothyroxine	Paroxysmal AF	4	2	Rivaroxaban	Surgery	4	Enoxaparin	PE	Enoxaparin, after 7 days phenoprocoumon	Hypercoagulable state
Nagasayi et al., 2017 [13]	88, F	Permanent pacemaker for SSS	N/A	AF	N/A	N/A	Rivaroxaban	Subdural hematoma after a fall	14	-	Upper extremity DVT	Reintroduction of rivaroxaban	Hypercoagulable state
Undas et al., 2017 [14]	32, F	Inherited thrombophilia	N/A	VTE	N/A	N/A	Rivaroxaban	HMB	2	-	3 days after end of menstruation DVT following the ankle injury	Enoxaparin, and later apixaban with reduction of HMB	Increased risk of VTE following irregular intake or withdrawal
	56, F	Inherited thrombophilia, hypertension, type 2 DM, COPD, obesity	N/A	VTE	N/A	N/A	Rivaroxaban	Pneumonia	7	Dalteparin	DVT	Enoxaparin, and later rivaroxaban	
Bakhit et al., 2016 [15]	65, F	Obesity, DM, hypertension, hypothyroidism, permanent pacemaker for TBS, stage 2 infiltrative ductal carcinoma of right breast 17 years ago	N/A	Paroxysmal AF	2	N/A	Rivaroxaban	RF catheter ablation	1	Heparin	Mobile septal aneurysm with thrombus	Aspiration of thrombus, UFH with warfarin	Hypercoagulable state
Turner et al., 2016 [16]	66, M	Hypertension, status post pericariocentesis	N/A	AF	N/A	N/A	Rivaroxaban	Pacemaker placement	1	-	Left MCA infarct	Transcatheter thrombectomy	Hypercoagulable state
									10	-	Worsening left MCA infarct, cerebral edema	Levetiracetam, dexamethasone ASA	
									11	-	TTE: Left atrial thrombus	Left atrial thrombectomy, later ASA and apixaban	
Weiler et al., 2014 [17]	84, M	State post abdominal surgery	N/A	AF	N/A	N/A	Dabigatran	Small bowel obstruction	2	-	UGIB, MI complicated by LVT with fatal embolization to the SMA	Exploratory laparotomy	Hypercoagulable state
Agarwal et al., 2013 [18]	65, M	Hypertension, hyperlipidemia	N/A	Left knee replacement surgery	N/A	N/A	Rivaroxaban	End of treatment	Ca. 2	-	MI with apical clot	PCI	Hypercoagulable state
											Next day SCA–PEA followed by VF	CPR	
											TTE: Pericardial effusion, LV wall rapture		
Finsterer et al. 2013 [19]	89, F	Hypertension, osteoporosis, osteochondrosis, spondylosis, anterolisthesis recurrent herpes genitalis, history of PE, MI, CI, status post vertebroplasty, aortocoronary bypass grafting, thyroidectomy, knee replacement, surgery for a rectocele	Levothyroxine, dronedarone, nicorandil, furosemide, pantoprazole, atorvastatin, lornoxicam, zolpidem, macrogol, isosorbide mononitrate, alprazolam	AF and status post TIA	N/A	N/A	Dabigatran	GFR < 30%	2	-	LGIB	2 blood transfusion	Prolonged anticoagulant effect, comedication
									5	-	Ischemic stroke	-	Hypercoagulable state
Stöllberger et al., 2013 [20]	80, M	Hypertension, hyperlipidemia, state post TURP	Furosemide, enalapril, allopurinol, hydrochlorothiazide, bisoprolol, simvastatin, amlodipine	AF	N/A	N/A	Rivaroxaban	Voluntary due to diarrhoea	5	-	Left MCA infarct	Physiotherapy, reintroduction of rivaroxaban after 4 weeks	Hypercoagulable state, dehydration

Abbreviations: AF—Atrial Fibrillation; ASA—Acetylsalicylic Acid; AT III—Antithrombin III; CI—Cerebral Infarction; CMO—Cystoid Macular Edema; COPD—Chronic Obstructive Pulmonary Disease; CPR—Cardiopulmonary Resuscitation; CRVO—Central Retinal Vein Occlusion; DAT—Device-Associated Thrombus; DM—Diabetes Mellitus; DVT—Deep Venous Thrombosis; F—Female; FLD—Fatty Liver Disease; HMB—Heavy Menstrual Bleeding; LAAOD—Left Atrial Appendage Occlusion Device; LGIB—Lower Gastrointestinal Bleeding; LMWH—Low-Molecular-Weight Heparin; LVT—Left Ventricular Thrombus; M—Male; MCA—Middle Cerebral Artery; MI—Myocardial Infarction; OAC—Oral Anticoagulant; PAD—Peripheral Artery Disease; PCI—Percutaneous Coronary Intervention; PE—Pulmonary Embolism; PEA—Pulseless Electrical Activity; RF—Radiofrequency; SCA—Sudden Cardiac Arrest; SMA—Superior Mesenteric Artery; SSS—Sick Sinus Syndrome; TBS—Tachycardia Bradycardia Syndrome; TEE—Transesophageal Echocardiogram; TIA—Transient Ischemic Attack; t-PA—Tissue Plasminogen Activator; TTE—Transthoracic Echocardiogram; TURP—Transurethral Resection of the Prostate; UFH—Unfractionated Heparin; UGIB—Upper Gastrointestinal Bleeding; VTE—Venous Thromboembolism.

**Table 2:** Synthesis of the findings from studies investigating the cessation of DOACs therapy and subsequent occurrence of prothrombotic rebound.

Authors and Year	Objective	Study Design	DOAC	Outcome	Intervention/Comparison	Key Findings
Álvaro Thomsen et al., 2024 [21]	To estimate association between DOACs discontinuation and risk of stroke among patients with NVAF	Retrospective cohort study	Apixaban, Rivaroxaban, Dabigatran, Edoxaban	Stroke	Discontinuation of DOACs; discontinuation 3 to 2 months vs. 6 to 3 months	DOACs cessation is associated with higher risk of stroke. Risk is the highest in the second and third months after cessation.
Mulholland et al., 2024 [22]	To evaluate the effect of inequalities in OAC prescribing by assessing SSE risk in people with AF	Retrospective cohort study	Apixaban, Rivaroxaban, Dabigatran, Edoxaban	SSE	OAC exposure: never started vs. continuous vs. discontinuous vs. cessation	SSE risk was significantly greater in those with discontinuous OAC compared with continuous. Cessation was associated with greater stroke risk than individuals that never started anti-coagulation and lower than individuals in the discontinuous OAC therapy cohort.
Cools et al., 2021 [23]	To investigate outcomes of patients with AF who discontinued OAC GARFIELD-AF	Prospective cohort study	N/A	SSE	Predictors of discontinuation; permanent vs. no discontinuation	Discontinuation for more than 7 days is associated with higher SSE, MI, and death risk.
Kim et al., 2021 [3]	To determine if stopping treatment induces a paradoxical rebound prothrombotic state	Observational	Dabigatran	Stroke	Short-term (1–3 days) vs. longer-term cessation	Short-term (3 day) cessation tended to be associated with relatively severity upon admission, compared with longer-term (>5 day) cessation.
Campello et al., 2020 [24]	To evaluate the VTE recurrence after anticoagulant discontinuation	Prospective cohort study	Rivaroxaban, Apixaban, Edoxaban, Dabigatran	VTE	DOACs vs. heparin/VKA discontinuation	DOACs associated with lower 2-year VTE recurrence risk than traditional anticoagulants after discontinuation.
Ando et al., 2019 [25]	To compare prothrombotic responses between the interrupted and uninterrupted therapies in patients undergoing cryoballoon ablation for paroxysmal AF	RCT	Apixaban	SSE	Uninterrupted vs. interrupted therapy	The uninterrupted therapy may decrease risk of hypercoagulability during the periprocedural period.
Park et al., 2018 [26]	To compare stroke outcomes between DOACs withdrawal in patient with NVAF	Retrospective cohort study	Rivaroxaban, Apixaban, Dabigatran	Stroke	Discontinuation of DOAC	Stroke that occurred after DOACs withdrawal was more severe at presentation and associated with poorer outcomes. The median interval between NOAC withdrawal and stroke was 7 days, which may indicate the occurrence of a rebound phenomenon.
Wagner et al., 2016 [27]	To ascertain the frequency of antithrombotic withdrawal and the risk of stroke	Survey Study	N/A	Stroke	Discontinuation of antithrombotic treatment	Survey did not investigate whether any strokes were a consequence of the discontinuation of antithrombotic treatment.
Yao et al., 2016 [28]	To evaluate how adherence to OACs affects the risk of stroke in patient with AF	Retrospective cohort study	Dabigatran, Rivaroxaban, Apixaban	Stroke	Adherence to OAC	Not observe excess risk shortly after treatment interruption.
Vanga et al., 2015 [29]	To study prevalence OAC discontinuation in patients with acute ischemic stroke	Retrospective cross-sectional cohort study	Dabigatran	Stroke	Discontinuation of OAC	About 2.6% or 1 in every 38 of all ischemic strokes occurred after OAC discontinuation. Strokes occurring after OAC discontinuation have higher mortality and morbidity.
Sherwood et al., 2014 [30]	To assess outcomes of temporary interruption in patients with AF ROCKET AF	RCT	Rivaroxaban	Stroke	Temporary interruptions of DOAC with LMWH bridging vs. no bridging	The data do not provide evidence to support or disprove the existence of rebound effect.
				Systemic embolism		
				MI		
Giugliano et al., 2013 [31]	ENGAGE AF-TIMI	RCT	Edoxaban	SSE	High-dose vs. low-dose	It is unlikely that there is a rebound activation of coagulation after edoxaban cessation.
Patel et al., 2013 [32]	To understand the possible risk of discontinuation inpatients with AF ROCKET AF	RCT	Rivaroxaban	SSE	Temporary interruptions vs. early permanent discontinuation vs. end of study transition	An increased risk of SSE after end of the study, importance of therapeutic anticoagulation coverage during a transition period.
Sairaku et al., 2013 [33]	To compare anticoagulant effects and safety during the AF ablation periprocedural period	RCT	Rivaroxaban, Dabigatran	D-dimer change	Rivaroxaban vs. Dabigatran	In comparison to dabigatran, rivaroxaban may potentially elevate the risk of hypercoagulability, a potential rebound effect, or a mismatch between half-life and dose.

Abbreviations: AF—Atrial Fibrillation; LMWH—Low-Molecular-Weight Heparin; MI—Myocardial Infarction; NVAF—Non-Valvular Atrial Fibrillation; OAC—Oral Anticoagulant; RCT—Randomized Controlled Trial; SSE—Stroke/Systemic Embolism; VKA—Vitamin K Antagonists; VTE—Venous Thromboembolism.

## Data Availability

All data were included and are available in the study.

## Abbreviations

AF	Atrial Fibrillation
aPTT	Activated Partial Thromboplastin Time
DOACs	Direct Oral Anticoagulants
dTT	Dilute Thrombin Time
ECA	Ecarin Chromogenic Assay
ECT	Ecarin Clotting Time
eGFR	Estimated Glomerular Filtration Rate
F1 + 2	Prothrombin Fragment 1 + 2
INR	International Normalized Ratio
NOACs	New Oral Anticoagulants
PRISMA	Preferred Reporting Items for Systematic Reviews and Meta-Analysis
PT	Prothrombin Time
TAT	Thrombin–Antithrombin Complex
TIA	Transient Ischemic Attack
TSOACs	Target-Specific Oral Anticoagulants
TT	Thrombin Time
